# The Gut in Early Life—Postnatal Challenges

**DOI:** 10.3390/children13040480

**Published:** 2026-03-30

**Authors:** Marc Alexander Benninga, Karl-Herbert Schäfer, Hugues Piloquet, Catherine Stanton

**Affiliations:** 1 Department of Pediatric Gastroenterology and Nutrition, Amsterdam University Medical Center, University of Amsterdam, Emma Children’s Hospital, Amsterdam, The Netherlands; 2 Working Group Enteric Nervous Systems (AGENS), University of Applied Sciences Kaiserslautern, Kaiserslautern, Germany; 3 Department of Pediatrics, University Hospital of Nantes, Nantes, France; 4 APC Microbiome Ireland, Teagasc Moorepark Food Research Centre, University College Cork, Cork, Ireland

**Keywords:** early life, healthy development, gastrointestinal tract, gut–brain axis, disorders of gut–brain interaction, gut microbiota, human milk, probiotic, prebiotics

## Abstract

The neonatal development period from the time of birth can be considered the period of greatest physiological changes throughout the human lifespan. These changes are partly due to dietary or environmental factors and are also modulated by genetic, neuronal, and humoral influences. The focus of research is increasingly on the microbial colonization of the neonatal intestine, since the establishment of a healthy, symbiotic newborn microbiota not only corresponds closely with nutrient metabolism, immune functions, and growth, but also with the brain as part of the so-called “gut–brain axis”. At the same time, a critical time window of opportunity opens up for the early infant microbiota, which is accessible to modulating approaches in favor of normal infant development. Although the definition of “normal” microbiota in infants still remains challenging, the microbiota of infants delivered at term can be discussed as the gold standard—provided they were exclusively breastfed and have not been exposed to antibiotics. Advances in sequencing technologies now also allow us to identify and characterize the microbiota at the strain level and to provide the scientific rationale for new approaches to modulate the early-life microbiome in a more targeted and personalized way—applicable also for formula-fed children who cannot be supplied with human milk. This review addresses the challenges associated with the “healthy” development of a newborn during the first weeks and months of life and discusses potentially modifiable external factors in light of the requirements for the establishment of a functional gut microbiota, gastrointestinal system, and gut–brain axis.

## 1. Introduction

### 1.1. A Critical Time Window for Neurological Development

In both the fetal and postnatal periods, a constant supply of nutrients is required in order to be able to maintain adequate healthy development and growth, in addition to vital body functions. The unique physiology of the newborn adapts quickly and constantly to extrauterine life and, in contrast to the adult physiology, is less stable and predictable [1]. Regarding the timeframe that is of most critical interest for children’s healthy growth, the timeframe of the ‘first 1000 days of life’ is often cited. This is also due to the fact that, between conception and the end of the second postnatal year, the most important neurological development processes take place during this period. Despite the lifelong continuation of neurodevelopment, it can be assumed that many of the processes that take place during this early phase cannot be repeated in a later phase of life. The expanded understanding of the complex interactions between micro- and macronutrients, as well as neurodevelopment, is seen as the key to an optimized nutrient supply during the child’s development. Due to the consequences that neurodevelopment has not only for an individual’s long-term health, but also for a person’s later productivity, the creation of an optimal perinatal nutritional environment is of fundamental socio-economic importance. The American Academy of Pediatrics (AAP) policy statement reaffirmed in October 2023 that improving nutritional health in the first 1000 days, with a focus on human milk, is an important factor for optimal nutrition and, thus, normal neurological development [2].

### 1.2. The Challenging Maturation Process of the Gastrointestinal Tract

**Figure 1:**
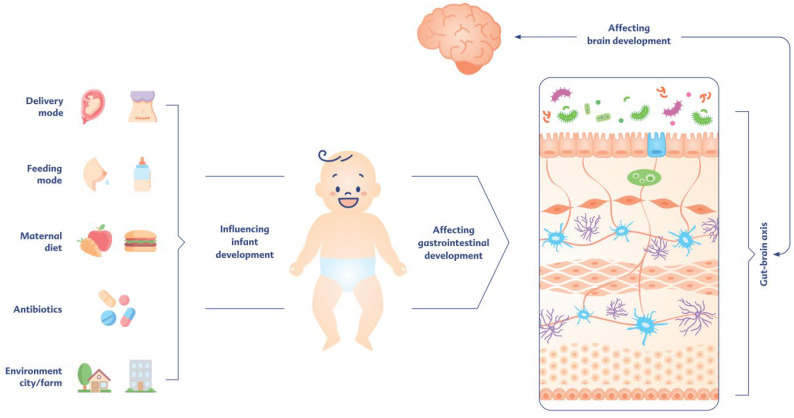
Perinatal and postnatal influences on gastrointestinal and brain development.

Anatomically and functionally, one of the largest surfaces that interacts with the environment through food intake, microbial colonization, and other potentially antigenic components in the human body is the gastrointestinal tract (GIT). From birth on, the newborn’s intestine experiences a series of maturation processes and adapts continuously to its environment and nutrition. As we know today, the GIT is not only relevant for digestion and absorption of nutrients but also fulfills neuronal, endocrine, exocrine, and immunological functions [3]. It should be mentioned at this point that the development of the GIT cannot be considered independently of healthy brain development during the early postnatal period. Even though gut maturation is not completely synchronized with brain development, there is a continuous bidirectional exchange mediated by a number of mediators and signaling pathways, for which the term ‘gut–brain axis’ has become established. According to this concept, the brain can modulate gastrointestinal functions such as motility and secretion, while conversely, the gut can send signals to the brain via immune, endocrine, systemic, and neuronal pathways [3,4]. There is also growing evidence that alterations that affect GIT development during the early postnatal period also affect brain development, and vice versa (Figure 1). Gut microbiota and its metabolic products, such as short-chain fatty acids (SCFAs), which are also established in the earliest stages of life, interact, for example, with the intestinal immune system, but they also have regulatory effects on the development, maturation, and functionality of microglia and brain-resident immune cells. Changes in the gut microbiota can therefore also affect processes that are important for brain development, such as the development of brain immunity and the permeability of the blood–brain barrier, as well as brain architecture and even neuronal circuits, as suggested by animal studies involving externally induced alterations of the maternal or postnatal microbiome [5].

The change in the absorption of nutrients via the amniotic fluid to the newborn’s gastrointestinal digestive system requires structural and functional maturity that enables the digestion and absorption of nutrients via colostrum and human milk [6]. The first indicator of normal GIT function within the first 24 to 48 h after birth is the meconium passage. Historically, the in utero environment and, consequently, the meconium were considered sterile; now, controversy exists whether a fetal microbiome develops during pregnancy [7]. While bacterial DNA in the meconium has been detected [8], Dos Santos et al. [9] demonstrated that the meconium does not harbor a resident microbial community that can be distinguished from the exogenous contamination introduced during sample processing and the sequencing workflow. However, looking at the meconium, it is a rich source of metabolites influencing microbiota diversity and maturation in early life [10]. In addition, other factors, such as mode of birth, genetics, and diet, are likely to determine the microbial colonization of the newborn [11,12].

The mode of delivery is considered a key factor influencing the composition of the postnatal infant gut microbiota, as the newborn’s gut is rapidly colonized by bacteria from the immediate environment—either from the mother’s vaginal and fecal microbiota or from the skin—depending on the mode of delivery. Furthermore, severely underweight, premature, and sick infants (delivered by cesarean section or vaginally) usually spend a considerable amount of time in the neonatal intensive care unit (NICU) after birth. The use of antibiotics before, during, and after delivery in mothers who deliver by cesarean section can further influence or disrupt the development of the gut microbiota [13]. One of the largest longitudinal whole-genome sequencing characterizations to date of the human gut microbiota in the previously little-studied neonatal period (≤1 month) has confirmed that the mode of delivery is an important factor for the composition of the gut microbiota in the first weeks of life and that the effect lasts until infancy (1 month to 1 year) [14]. However, it remains challenging to determine the specific influence of a delivery mode on the development of the microbiota over the course of the child’s life or on the child’s later health. There are also studies suggesting that the microbial composition may recover over time and be only temporary, without the effects on the child’s outcome being fully understood [15].

Adequate development of the intestinal microbiota of the newborn is important for, e.g., the prevention of osmotic diarrhea and the synthesis of relevant biologically active metabolites. Moreover, in the long term, and with regard to neurocognition, proper development of the immune system—including mitigating susceptibility to, for example, infections and atopic or metabolic diseases and ensuring general well-being—is an indispensable requirement [3,16,17].

Despite the fundamental importance of the implications these transformation processes have on the healthy development of the child, comparatively little is known about the complex developmental paths of digestive functions. Their research is made more difficult by the required invasiveness of the examinations in healthy term infants. Most of the information we currently have was obtained from preterm infants with an immature gastrointestinal tract, whose nutrient supply must be ensured via the nasogastric tube [6]. There are also experimental animal data that provide a more precise picture of the developmental milestones in the GIT during the perinatal period, critical for the absorption of orally administered pharmaceutical products. A comprehensive review of human and animal experimental data involving the US Food and Drug Administration [18] indicates how variable the maturation processes in the intestine are, even in term newborns, and how they depend on many factors (including fed or fasted state, type of food, hormones, and intestinal microbiome). Several characteristics can be taken into account, including the increased absorption capacity of nutrients in the neonatal colon (not observed in adults), macromolecular absorption (e.g., with transplacental immunoglobulins and via human milk), the initially elevated gastric pH value, the metabolism in terms of biotransformation and conjugation reactions, the neonatal microbiome, the gastric emptying times, and excretion through the feces. In contrast to earlier assumptions, for mean intestinal transit times, there are recent data that indicate fewer differences between healthy newborns/infants and adults [18]. However, during the developmental changes in the first weeks and months of life, gastrointestinal symptoms may occur, causing distress for infants and parents.

## 2. Establishing the Link Between the Gut and the CNS

### 2.1. Where Does the Interplay Between the Gut and the Brain Take Place?

The early development of the brain is codependent on the appropriate functioning of other organ systems, in which the GIT is likely to play the largest role [19]. The metabolic demands alone during the early development phase of the brain can be enormous, especially since the brain requires up to 20% of the total body energy in homeostasis, although it only accounts for 2% of the body weight. The newborn’s brain grows from 36% to 80–90% of adult volume in the second year of life. This is accompanied by a massive expansion of the central nervous system (CNS) due to the establishment of relevant connections and wiring, including maturation of neuronal fiber tracks and synaptogenesis. Interestingly, it has been demonstrated that a normal gut microbiota is an important prerequisite for the healthy development of the CNS [20].

As introduced above, the bidirectional relationship between the GIT and the brain or the CNS is also referred to as the ‘gut–brain axis’ or ‘microbiota–gut–brain axis’. In part, communication is mediated by the vagus nerve and its connections to the enteric nervous system (ENS). As the largest and most complex part of the peripheral nervous system, the ENS is found throughout the whole GIT, is closest to the gut microbiota, and works independently of the brain. From an evolutionary point of view, the ENS can even be referred to as the “first brain”, given the evidence that the CNS developed successively after the evolution of an ENS-like nervous system. As the ENS is considered a key modulator of gut barrier function and regulator of enteric homeostasis, impaired integrity of the intestinal wall can lead to bacteria and their metabolites, as well as food components, being able to migrate and trigger chronic inflammatory reactions [21].

**Figure 2:**
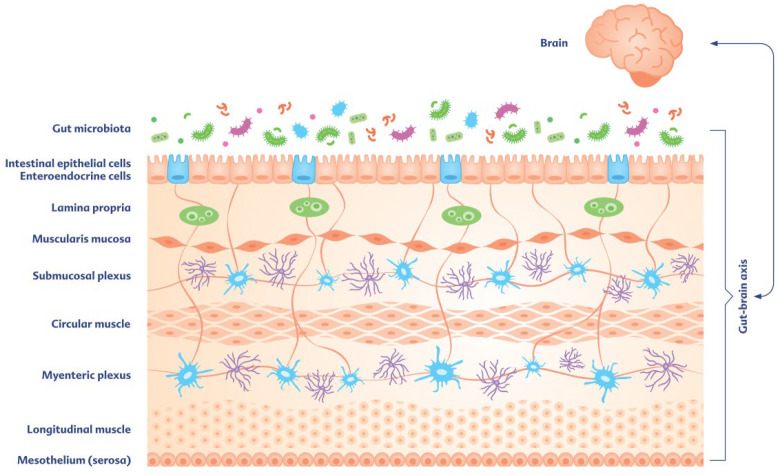
The gut–brain axis—a bidirectional interplay between the gut and the CNS. The ENS regulates a variety of gastrointestinal functions and communicates bidirectionally with the brain. This schematic overview is a highly simplified representation of the laminar structure of the intestine. The two main plexuses of the ENS are represented by the myenteric plexus, located between the circular and longitudinal muscle layers of the muscularis externa, and the submucosal plexus in the submucosa. This reciprocal relationship within the gut–brain axis is modulated by the presence and activity of the gut microbiota, whose signaling also functions bidirectionally. The communication network between the gut, microbiome, and CNS becomes significantly more complex when—as not shown graphically here—communication via multiple neuronal circuits and a multitude of different enteral neuron subtypes, the autonomic nervous system, and neuroimmunological and neuroendocrine signaling pathways are taken into account, which include, for example, hormones, humoral signaling molecules, and metabolic products of the microbiome.

Embedded within different layers of the intestinal tract, the ENS is organized into distinct networks within the gut wall, with two major ganglionated networks—including the myenteric plexus and the submucosal plexus (Figure 2). The myenteric plexus is located between the longitudinal and circular muscle layers, facilitating peristalsis, while the submucosal plexus is located between the circular muscular and mucosal layer, coordinating secretion and neuroimmune interactions [22]. The ENS contains >100 million neurons with many different subtypes, including, for example, intrinsic primary afferent neurons (IPANs), considered as one of the main classes into which the neurons of the submucosal plexus can be categorized [23]. Thus, the ENS has more neurons than all other peripheral ganglia combined and at least as many as the spinal cord. The phenotypic diversity of enteric neurons is vast, with virtually every neurotransmitter class found in the CNS also present in the ENS. Given that 90% of vagal fibers between the gut and brain are afferent, the brain is likely more of a receiver (e.g., for signals that trigger central vasovagal reflexes or the mediation of sensations such as satiety) than a transmitter in gut–brain communication.

Since survival depends on the functioning of the ENS at birth to enable oral feeding, the ENS circuits necessary for motility must be developed before birth [24]. It is known that enteric neurons, depending on their stage of development in the cell cycle (e.g., serotonergic early enteric neurons), perform distinct regulatory functions in brain-related neurogenesis—with corresponding consequences if this step is disrupted. In children with irritable bowel syndrome (a functional disorder characterized by impaired intestinal motility and visceral pain), signs of serotonergic dysfunction have been observed in the intestinal mucosa, which could also affect concurrent neuronal development [23]. This review does not aim to discuss the extensive findings on the neural development of the ENS and its implications for the brain in depth, but interested readers can refer to excellent previously published articles for further information [23].

The development of the ENS, along with that of the intestinal barrier, intestinal motility, and mucosal enteric glial cells, apparently continues after birth [25]. Postnatal development is also considered a crucial phase for establishing the gut microbiome and consequently shaping gut health [26]. However, while the molecular and cellular mechanisms that control the development of the ENS during fetal growth have been studied intensively, postnatal gut development is an emerging field of research. Furthermore, the understanding of the gut–brain axis during the co-development of the GIT and brain in the early postnatal phase is still limited [25]. In principle, brain development in the prenatal phase seems to be mainly influenced by genetic determinants, while in the early postnatal phase, environmental factors become more important, either promoting or hindering brain development [19].

For example, it has been shown that the microbiota has an effect on the intestinal epithelium and, consequently, on the production of serotonin by enterochromaffin cells, absent in a germ-free environment. In addition, in germ-free mouse models, the number of neurons and the composition of neurons were changed, and the frequency and amplitude of muscle contractions decreased in the ileum and jejunum. Therefore, it has been suggested that the microbiota in early postnatal life is crucial for the development of enteric neurons and intestinal contractility [27,28,29,30].

In postnatal myenteric plexus cultures, human milk proteins promoted neurogenesis and neurite outgrowth [31]. Feeding mice a prebiotic-supplemented high-fat diet resulted in fructo-oligosaccharides reducing ex vivo duodenal contraction frequency, despite the fact that the oligosaccharides were fermented in the colon. The results suggest that prebiotics can have an impact on gastrointestinal motility [32]. Furthermore, specific bacteria of the microbiota produce metabolites like gamma-aminobutyric acid (GABA) or short-chain fatty acids (SCFAs), which can affect gut motility. As part of the microbiota, certain lactobacilli can attenuate stress-induced gut motility changes, playing an important role in ENS development, while supporting the postnatal establishment of the ENS [33]. A disruption of the microbiota through antibiotic exposure could affect the physiology of the gut as well as the ENS. The treatment of neonatal mice with vancomycin had lasting effects on the activity of enteric neurons, as well as lasting effects on gastrointestinal microbiota diversity and composition [34]. The transition from liquid to solid food during weaning represents another significant environmental change, which coincides with a further phase of ENS maturation. During this time, significant changes in synaptic contacts and neurochemistry have been observed. This also applies to the vagus nerve, which is not yet fully myelinated at birth. From the 32nd week of gestation until the 6th month of life, an accelerated myelination rate can be observed, which may be related to the supply of nutrients provided by human milk that are important for the myelination process [19].

However, the impact of early-life nutrition on the microbiota and the nervous system in the GIT is complex and requires further research to understand possible implications for a growing infant and its gut maturation. Many insights have been gained through studies on germ-free mouse models. These have provided important evidence for the assumption that the microbiome is involved in brain development processes related to stress hormone signaling, neuronal function, and neuroprotection; however, there are limitations regarding the generalizability of these results to humans [35].

### 2.2. Disorders of Gut–Brain Interaction—Paradigmatic for a Dysfunctional Gut–Brain Axis?

Recently, the former term “functional gastrointestinal disorders” (FGIDs) was renamed to “disorders of gut–brain interaction” (DGBIs) to underline the importance of gut–brain interaction in frequently observed phenomena of the first months of life, such as regurgitation, infant colic, functional constipation, infant dyschezia, cyclic vomiting syndrome, rumination, and functional diarrhea. For parents, the concept of a disturbed gut–brain axis is easier to convey than the older term “FGIDs”, which could also be seen as potentially stigmatizing because of the use of “functional”; the former term also indicates a medically inexplicable, non-specific genesis [36,37]. According to the Rome IV classification, these disorders represent a variable combination of chronic or recurrent symptoms that cannot be traced back to underlying structural or biological abnormalities [38]. Although there are no exact incidence data for DGBIs between birth and the 3rd year of life, functional regurgitation and infant colic appear to be the most common DGBIs in the first months of life, disappearing in the following months [39,40]. In a cross-sectional, multicenter study in Belgium, Italy, and the Netherlands including 2751 children, infant regurgitation (13.8%) occurred most frequently in infants, and functional constipation occurred most frequently in toddlers (9.6%). According to Rome IV criteria, in infants aged 0–12 months, the prevalence of “any DGBI” was more than twice as high (24.7%) as in toddlers aged 13–48 months (11.3%). Multivariable regression analyses revealed that younger age and formula feeding were associated with the prevalence of any DGBI among infants [41]. Despite the transient and benign course in most patients, DGBIs have been associated with adverse sequelae, such as shortened duration of breastfeeding and short- and long-term effects on behavior, food intake, and sleep [39]. Furthermore, several problems can also arise, such as disturbed parent–child interactions, anxiety and distress, abuse of medication, and high use of healthcare resources, which become entrenched and result in a vicious circle [42]. It is also known that DGBIs can develop progressively in childhood, extending into adulthood and possibly leading to, for example, irritable bowel syndrome [43].

The Rome criteria were developed by the working committees of The Rome Foundation and were initially only applicable to adults. However, this changed with the Rome II criteria, and from the Rome III criteria onwards, a distinction was made between younger (infants/toddlers) and older children. Due to the improved data on epidemiology, pathophysiology, and diagnostic procedures, the evidence base for the most recent version of the criteria (Rome IV) has increased, and the diagnostic criteria appear to be more specific overall [44]. This allows for an approach that avoids unnecessary examinations to rule out organic causes, causing less stress for patients and relatives [45]. In particular, major changes have been made to the Rome criteria for infant colic [44]. Wessel et al.’s previous “rule of three” criteria (crying more than 3 h a day, for more than 3 days a week, for more than 3 weeks in a row) [46] have been replaced by criteria that focus more on the factors that cause distress in parents. Accordingly, diagnosis of infant colic requires (1) an age of <5 months at onset/cessation of symptoms; (2) recurrent and prolonged periods of infant crying, fussing, or irritability reported by caregivers that occur without obvious cause and cannot be prevented or resolved by caregivers; and (3) no evidence of infant failure to thrive, fever, or illness. The Rome IV criteria point out that, despite the lack of a confirmed DGBI, infants with colic are often referred to pediatric gastroenterologists [47].

### 2.3. What Measures Promote the Well-Being of Infants (And Parents)?

It should also be taken into account that the frequency and pattern of crying vary depending on age and changes during the first months of life, without it necessarily being pathological. Despite all the deviations observed, there is a “normal crying curve” in infants. An increased duration of crying is therefore expected in the first weeks of life, followed by a decrease and stabilization by week 12. Since this pattern cannot be influenced by the type of care, it is assumed that it is a physiological and necessary part of neurobehavioral development. Even if infant colic is mostly benign and self-limiting, the excessive crying is very distressing for family members and caregivers [12]. The currently discussed explanations for the largely still unknown causes underlying infant colic include intestinal dysbiosis (resulting in increased intestinal pain sensations, as well as successive pro-inflammatory changes in the intestine), insufficient bile acid production (due to immature enterohepatic circulation), and ENS immaturity (facilitated by the microbiota–gut–brain axis), which may lead to an adverse pattern of intestinal motility and sensory functions. Other possible gastrointestinal factors that are repeatedly discussed in the genesis of infant colic include food allergy (especially to cow’s milk proteins), dysmotility, psychosocial factors (e.g., parental stress, abnormal parental and infant coping behavior), and feeding mode (e.g., formula feeding) [12,39]. Caregiver’s anxiety and distress can unintentionally lead to a negative feedback loop, which intensifies the emotional reactions of both the parents and the infant. The not yet fully developed gut–brain axis could further intensify this effect, with emotional distress manifesting as a DGBI [48]. There appears to be a constant bidirectional dialogue between the brain and the gut, with preclinical studies suggesting that changes in the gut microbiota may influence brain signaling pathways related to pain and associated emotional behavior [49]. Conversely, the maternal psychological well-being appeared to reduce the risk of infant colic, as suggested by a cross-sectional study of mothers in the first postpartum year [50].

**Figure 3:**
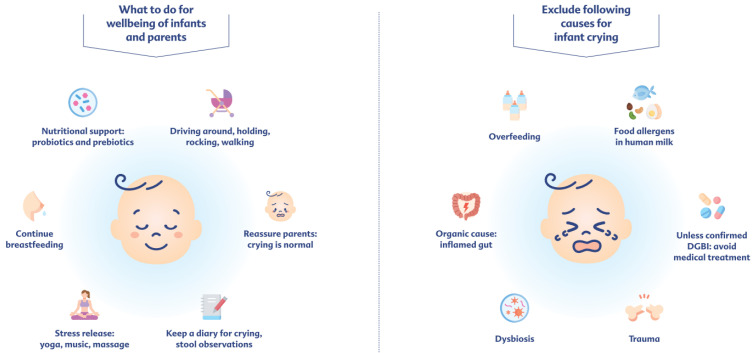
Suggestions to consider for the well-being of infants and parents.

Depressive or anxiety-related symptoms in parents should also be recognized and addressed. However, there is currently no clear evidence that the manifestation of infant colic in particular is caused solely and independently by external factors such as the mode of birth, prenatal maternal stress, the administration of antibiotics to the infant/the mother’s use of medication, environmental factors at home and in the hospital, or the infant’s feeding method [12]. Therapy management of infant colic is currently largely not evidence-based. Unless there are signs of an underlying serious organic cause, reassuring and supporting the parents is one of the measures encouraged (Figure 3). This can include informing parents about signs of hunger and fatigue, about the transient nature of DGBIs, and about the importance of regular daily structures, as well as tips such as holding the child [42]. Parents should also be made aware that DGBI-related symptoms in the first few months of life are transient and mostly resolve spontaneously [12]. Nutritional advice is already one of the leading principles of treatment for children with DGBIs, e.g., in regard to the frequency of meal intake, their quantity, the avoidance of overfeeding and excessive fluid intake, the encouragement for breastfeeding, or the correct preparation of milk formula for children who are not breastfed [39]. Furthermore, it is increasingly becoming apparent that the role of the microbiome is of particular interest in pathophysiology and as a potential approach for dietary interventions [12]. Therefore, parents should also be informed about nutritional interventions to be considered in the case of persistent DGBI symptoms with proven effects on formula-fed infants.

## 3. The Brain–Gut–Microbiota Axis: Gut Bacteria Are Needed for Healthy Development

### 3.1. The Dynamics of the Early-Life Intestinal Microbiota

The previous considerations suggest that the infant microbiome is in a symbiotic exchange with its host in order to fulfill various tasks—not least in relation to optimal neurodevelopment [51]. Therefore, establishing a healthy and homeostatic symbiotic relationship is of crucial importance for many developmental processes, including one particular highly dynamic process [52], responding to dietary changes. In this regard, in particular, the first 12 months of life are considered an important time period to positively influence the intestinal microbiota and thus the further individual development through interventions such as nutritional supplementation [53,54].

The human gut microbiota comprises up to around 100 trillion microorganisms in the GIT [17,55], whose gene repertoire far exceeds the human genome. Neither the exact time at which microbial colonization of the intestinal tract begins nor the time at which development is largely “complete” and corresponds to the adult microbiome is known. However, the finding that the diversity of the microbiota increases up to the 4th year of life has been well documented [56]. The origin of the child’s microbiome comes from several sources, including vertical mother–child transmission as the initial largest and most important milestone.

In the first 24 h after birth, the microbial colonization is still low in complexity, with a single phylum limited to three to seven species. As early as the following month, the diversity of the infant microbiota in the GIT increases, and, especially with the intake of breast milk, the abundance of initially observable facultative microbes such as *Enterobacteria*, *Escherichia* or *Shigella* decreases. Instead, the abundance of anaerobic bacteria, especially *Bifidobacterium* and *Bacteroides*, increases; the latter have been proven to be dominant in the infant gut. Between month 1 and 6, Actinobacteria (mainly *Bifidobacterium* that can utilize human milk oligosaccharides) dominate the infant’s gut microbiota, and this effect has been observed to take place regardless of delivery mode [57]. In children aged 3 months of age and above, microbial profiles have been shown to develop over time, and they can be broadly divided into three phases: an initial phase in which all 5 phyla undergo significant changes (*Actinobacteria*, *Firmicutes*, *Verrucomicrobia*, *Proteobacteria*, and *Bacteroidetes*), a transitional phase in which mainly two phyla undergo significant changes (*Proteobacteria* and *Bacteroidetes*), and a stable phase in which all phyla remain unchanged [58]. From the age of 6 months on, which roughly corresponds to the end of exclusive breastfeeding, the introduction of solid food again leads to significant changes, including a reduction in *Bifidobacterium* [59]. However, these models primarily reflect infants growing up in industrialized countries, and they are not necessarily generalizable [60]. Approximately 25–30% of infant gut microbiota is derived from human milk [61]. Microorganisms contained in human milk seem to be metabolically active in the infant gut, metabolizing the oligosaccharides present in human milk to SCFAs. Preclinical studies suggest that certain isolates from human milk may be bacteriostatic and/or might have a bactericidal effect. Although the role of breastfeeding in shaping the infant gut microbiota is the best studied to date, it is likely that other niche microbiota systems—such as the oral, nasopharyngeal, and respiratory microbiota—might also play an important role [62].

Understanding of what makes a healthy microbiome remains limited, and relevant research is ongoing. The composition, diversity, and functionality of the microbes are subject to a high level of variation; thus, it hardly seems possible to define a generally valid standard for the “normal” development of an early intestinal microbiome. The establishment, development, and stabilization of the gut microbiota depend on multiple intrinsic and extrinsic factors, including mode of delivery. As mentioned before, a frequently cited example of the interaction between the infant microbiome profile and the delivery mode is represented by those born by cesarean section (CS delivery), who, in contrast to infants born vaginally, experience their first colonization mostly through the skin and from the environment. The gut microbiome in those born by elective CS was associated with lower diversity [63]. The establishment of the maternal and infant microbiota has also been shown to be affected by the diet(s) that the mother follows during pregnancy [64]. Intestinal dysbiosis, in the sense of lower diversity of the gut microbiota and differences in microbiota, was observed more frequently in infants with colic during the first two weeks of life than in age-matched control infants without colic, resulting in excessive crying behavior [65], suggesting close communication along the microbiota–gut–brain axis. To adequately shed light on the interplay between the gut microbiota and ENS or brain, further clinical studies that systematically address possible connections between microbiota, nutrition, and CNS development, especially in humans, are needed [66]. At the very least, murine studies have shown that germ-free mice exhibit behavioral abnormalities in the early postnatal phase, which normalize with early microbial colonization of the gut. Possible neurochemical causes at the brain level include regionally varying expression of receptors for gut hormones such as 5-hydroxytryptamine (5-HT); increased turnover of neurotransmitters such as norepinephrine, dopamine, and 5-HT; and differing levels of proteins with regulatory effects on the development of neuronally relevant structures [30].

### 3.2. Human Milk—The Ultimate Developmental Food

Human milk offers the best benefits and is the gold standard for balanced infant nutrition [64]. Breastfeeding not only provides essential nutrients for infants but also contributes to direct and indirect protective mechanisms against infections of the respiratory and gastrointestinal tracts, as well as inflammatory diseases [57,67]. The World Health Organization (WHO) and international medical societies therefore recommend that newborns should be breastfed exclusively for at least six months, followed by the introduction of complementary food [62].

Historically viewed as a sterile liquid, it is now known that human milk has a complex and diverse microbial composition—again, with a highly variable inter- and intra-individual profile. For example, there is growing evidence for the presence of, among many other components, *Lactobacillus*, some *Bifidobacterium*, *Staphylococcus*, *Streptococcus,* and *Enterococcus* [62].

In addition to the microorganisms, human milk oligosaccharides (HMOs) play a role in the bioactive composition of human milk. HMOs are complex glycans which quantitatively make up a large proportion of human milk. In general, their concentrations in human milk are higher than in the milk that can be obtained from domesticated animals. HMOs apparently serve as prebiotic glycans for the growth of beneficial microorganisms. Studies suggest that HMOs also play a role in preventing infections, promoting intact epithelial and mucosal barrier maturation and function, and ensuring adequate immune cell function. In particular, they support the growth of *Bifidobacterium*, which cannot be found in comparable numbers in formula-fed infants, as can be seen in the example of *Bifidobacterium longum* subspecies *infantis*, which can utilise numerous HMOs and is enriched in the microbiota of breastfed infants [68].

Another research field that is currently being intensively investigated is the metabolomics of human milk, and this is also due to improvements in analytical technology. Thanks to the INFAMILK study, which was established in Cork, the Republic of Ireland, it is now possible to show how the human milk metabolome dynamically develops in term infants. New data on changes in metabolite concentrations during early and late lactation were also obtained. Certain metabolites show higher levels during early lactation (e.g., oligosaccharides, cis-aconitate, O-phosphocholines, O-acetylcarnitines, gluconate, and citric acid), as well as higher levels of lactose, 3-fucosyllactose, glutamine, glutamate, and short- and medium-chain fatty acids during later lactation [69,70].

### 3.3. Probiotics in Infant Colic—What Have We Learned?

For various reasons, not all newborns can benefit from breastfeeding. An estimated 19% of infants do not receive human milk during infancy, and only 22% of breastfed infants receive human milk exclusively for the recommended period of 6 months [2]. Given the above-mentioned advantages of a “healthy” microbiota, there is growing interest in perinatal dietary interventions for infants who cannot be optimally breastfed but are amenable to microbiota modulation. This also includes the use of probiotics, prebiotics, and synbiotics or postbiotics in infant formulas during infancy [64,71].

According to the WHO’s 2014 definition, probiotics are defined as “live microorganisms that, when administered in adequate amounts, confer health benefits on the host” [72]. As previous studies suggest, probiotic supplementation has the potential to shift the microbial profile in favor of beneficial colonizing microbes. This may enable us to counteract the overgrowth of, for example, gas-producing coliforms and may activate anti-inflammatory and regulatory immune effects in the settings of, for example, mucosal inflammation [73,74], while also likely promoting intestinal homeostasis, in particular via the modulation of permeability and peristalsis, with possible beneficial effects on the gut–brain axis and regulation of hypersensitivity [75].

For infants who cannot be breastfed, enrichment of infant formula with some of the unique components limited to human milk (e.g., cytokines, growth factors) does not currently appear to be promising for reasons related to stability and high costs. Rather, supplementation with beneficial health-promoting prebiotics, probiotics, or their combination in the form of a synbiotic could offer a rational and pragmatic approach for expanding the range of infant formulas [76]. Preclinical study data indicate a beneficial influence on the immune system, as well as antimicrobial effects, improved barrier integrity in intestinal epithelial cells (e.g., by promoting mucin expression), bifidogenic effects (increasing the abundance of bifidobacteria), and an increased abundance of microbes that produce metabolites with beneficial properties and reduce intestinal dysbiosis [77,78,79].

The spectrum of probiotics tested as a supplement to infant formula includes, among others, *Streptococcus thermophilus*, *Limosilactobacillus reuteri* (formerly *Lactobacillus reuteri* DSM 17938), *Bifidobacterium breve*, *Bifidobacterium lactis* Bb12, *Limosilactobacillus fermentum* CECT5716 (formerly *Lactobacillus fermentum* CECT5716), and *Lacticaseibacillus rhamnosus GG* (*formerly Lactobacillus rhamnosus GG*) [80]. The most frequently evaluated prebiotics in published clinical trials are fructo-oligosaccharides (FOSs), galacto-oligosaccharides (GOSs), and human milk oligosaccharides (HMOs) [71].

Looking at DGBIs in infants as one of the most common challenges in daily primary care practice, dietary modifications with the supplementary use of probiotic microorganisms offer a promising approach, especially due to the fact that they have shown no or only minor adverse effects [75,81]. A systematic review of studies and clinical guidelines published between 2009 and 2019 for the most common treatment approaches in infant colic revealed the strongest available evidence for the use of probiotics. Of 32 identified studies, 12 studies addressed the use of probiotics, including 10 meta-analyses or narrative evaluations of several studies. The authors concluded that there is high-level evidence that probiotics (particularly *L. reuteri*) are most effective in reducing crying time (range: minus 25 to minus 65 min over 24 h) in breastfed children [81]. A Cochrane analysis, which was also included in the systematic review mentioned above, evaluated randomized controlled trials in newborn infants less than one month of age and without infantile colic in terms of prophylactic effects (preventing or reducing severity of infant colic). Although there was no evidence that probiotics are more effective than placebo in preventing infantile colic, the use of probiotics was associated with reduced crying time compared to placebo. There were no differences in the side effects observed [82].

For other dietary interventions, the data appeared to be more limited. A Cochrane review from 2018 came to the conclusion that the evidence for individual dietary modifications is still insufficient to be able to make specific recommendations. Studies involving probiotics were not included in this analysis [83]. Compared to the data available on the use of *Limosilactobacillus reuteri* DSM17938 in breastfed infants with colic, the evidence in favor of other probiotic strains concerning DGBIs is currently insufficient [39,84].

Beneficial effects have been shown in infants receiving a follow-on formula enriched with *L. fermentum* (CECT5716). At the end of a 6-month study, the incidence rate of gastrointestinal infections in the experimental arm was reduced by 46% (*p* = 0.032), and the incidence of upper respiratory tract infections was significantly reduced by 27% (*p* = 0.026) compared to the control group [85]. In a more recent clinical trial, the synbiotic combination of *L. fermentum* (CECT5716) and GOS in an infant formula did not demonstrate an effect on gastrointestinal infections when compared to control formula-fed infants. However, feeding with the synbiotic formula resulted in a significant 23% reduction in lower respiratory tract infections over the first year of life. The authors suggested that this effect could have been due to gut microbiota maturation and activation of the gut immune system [86]. The synbiotic formula indeed significantly increased the occurrence of *Bifidobacterium* spp. and *Lactobacillaceae* and lowered the occurrence of *Blautia* spp., as well as *Ruminoccocus gnavus* and relatives, compared to the control infant formula at 4 months of age. Conducting *de novo* clustering at 4 months of age, the overall phylogenetic profiles of the infants receiving the synbiotic formula were closer to a reference microbiota profile of those fed with human milk than those fed the control formula [87].

Therefore, further clinical trials are needed to provide more specific recommendations for the use of probiotics in formula- or breast-fed infants who are at increased risk of developing DGBIs. Clinical studies conducted with probiotics often show high clinical heterogeneity (e.g., in terms of the range of strains used, dosages, and routes of administration, as well as in the study populations included), which makes it difficult to compare study results. Preterm and term infants were frequently mixed, and breast and bottle feeding were not clearly assessed separately. The timing of probiotic administration also varied (administered before and after delivery to the breastfeeding mother, or to the mother before delivery and to the infant after birth) [82]. Suitable studies will include a sufficient number of participants (previous studies often have small sample sizes) to allow for statistically significant conclusions to be drawn, and future studies should also clearly define diagnostic inclusion and outcome criteria as part of standardized research protocols.

Treatment with pharmacological agents is generally not supported (with the exception of laxatives for functional constipation), since evidence of the benefit of pain-relieving agents, simethicone, prokinetic drugs, and acid suppressive agents in DGBIs in infants and toddlers is limited/lacking [12,39,88].

## 4. Conclusions

• The first 1000 days of life mark the crucial time window for setting the course for the child’s healthy neurodevelopment. It is increasingly recognized that the composition of the microbiome can influence neurodevelopment—for example, by influencing the ENS—and that a healthy microbiome supports the metabolic requirements for healthy brain development (gut–brain axis).
• A common phenomenon that is closely related to the healthy development of the gut–brain axis is the occurrence of gastrointestinal symptoms that cannot be explained by structural or biochemical abnormalities (disorders of gut–brain interaction (DGBIs)). There is evidence of a complex interplay between factors such as intestinal dysbiosis, ENS maturation, the mucosal intestinal barrier, and feedback mechanisms within the microbiota–gut–brain axis or psychosocial stress. However, it remains a key concern to educate parents and caregivers that infantile colic is a benign condition which usually does not require treatment.
• Supportive approaches that contribute to a healthy microbiome and have beneficial effects on a disturbed gut–brain axis, as well as dietary recommendations including probiotic and/or prebiotic supplementation, may be of interest. However, further evidence is needed to recommend them for systematic use.
